# Biomarkers of Seizure Activity in Patients With Intracranial Metastases and Gliomas: A Wide Range Study of Correlated Regions of Interest

**DOI:** 10.3389/fneur.2020.00444

**Published:** 2020-05-29

**Authors:** Piyush Kalakoti, Alicia Edwards, Christopher Ferrier, Kanika Sharma, Trong Huynh, Christina Ledbetter, Eduardo Gonzalez-Toledo, Anil Nanda, Hai Sun

**Affiliations:** ^1^Department of Neurosurgery, Louisiana State University Health Science Center, Shreveport, LA, United States; ^2^Department of Neurology, University of Iowa Hospitals and Clinics, Iowa City, IA, United States; ^3^Department of Neurosurgery, Robert Wood Johnson Medical School, New Brunswick, NJ, United States; ^4^Department of Neurosurgery, Rutgers University, Newark, NJ, United States; ^5^Neuroradiology, Department of Radiology, Louisiana State University Health Science Center, Shreveport, LA, United States

**Keywords:** gliomas, intracranial metastases, seizures, pars orbitalis, supramarginal gyrus, pre-cuneus, brainsuite, temporal plus epilepsy

## Abstract

**Introduction:** Studies quantifying cortical metrics in brain tumor patients who present with seizures are limited. The current investigation assesses morphometric/volumetric differences across a wide range of anatomical regions, including temporal and extra-temporal, in patients with gliomas and intracranial metastases (IMs) presenting with seizures that could serve as a biomarker in the identification of seizure expression and serve as a neuronal target for mitigation.

**Methods:** In a retrospective design, the MR sequences of ninety-two tumor patients [55% gliomas; 45% IM] and 34 controls were subjected to sophisticated morphometric and volumetric assessments using BrainSuite and MATLAB modules. We examined 103 regions of interests (ROIs) across eight distinct cortical categories of interests (COI) [gray matter, white matter; total volume, CSF; cortical areas: inner, mid, pial; cortical thickness]. The primary endpoint was quantifying and identifying ROIs with significant differences in z-scores based upon the presence of seizures. Feature selection employing neighborhood component analysis (NCA) determined the ROI within each COI having the highest significance/weight in the differentiation of seizure vs. non-seizure patients harboring brain tumor.

**Results:** Overall, the mean age of the cohort was 58.0 ± 12.8 years, and 45% were women. The prevalence of seizures in tumor patients was 28%. Forty-two ROIs across the eight pre-defined COIs had significant differences in z-scores between tumor patients presenting with and without seizures. The NCA feature selection noted the volume of pars-orbitalis and right middle temporal gyrus to have the highest weight in differentiating tumor patients based on seizures for three distinct COIs [GM, total volume, and CSF volume] and white matter, respectively. Left-sided transverse temporal gyrus, left precuneus, left transverse temporal, and left supramarginal gyrus were associated with having the highest weight in the differentiation of seizure vs. non-seizure in tumor patients for morphometrics relating to cortical areas in the pial, inner and mid regions and cortical thickness, respectively.

**Conclusion:** Our study elucidates potential biomarkers for seizure targeting in patients with gliomas and IMs based upon morphometric and volumetric assessments. Amongst the widespread brain regions examined in our cohort, pars orbitalis, supramarginal and temporal gyrus (middle, transverse), and the pre-cuneus contribute a maximal potential for differentiation of seizure patients from non-seizure.

## Introduction

Seizures are a common neurological symptom that can be provoked by toxins, head trauma, electrolyte imbalances, brain hemorrhage, and/or tumors (1). Among brain tumor patients, seizures are often the only presenting clinical symptom, and its incidence varies across different tumor histopathology (2–5). The presence of seizures in tumor patients impacts the quality of life and is associated with worse outcomes following surgical resection (4, 5). Low-grade gliomas (LGGs) tend to have a higher estimated incidence of seizures, ranging from 60–75% (6–11), compared to high-grade gliomas (HGGs) [25–60%] (12–14) or intracranial metastases (IM) [20–35%] (15, 16). The variation in seizure incidence is primarily linked to tumor size, location (8), and possibly to the tumoral and peritumoral microstructure. A comprehensive understanding of these microarchitectural variations can potentially aid our ability to predict seizures and subsequently serve for implementing treatments to prevent seizures in tumor patients before its onset. In the contemporary era of cost-containment ushered by the introduction of bundled payments, such efforts could improve the value in neurosurgical healthcare delivery in tumor patients with seizures.

Studies have characterized the anatomical locations of HGGs to areas with a high ratio of gray and white matter volume, while the LGGs tend to have a preferential bias toward the secondary functional regions of the brain (17, 18). Our recent investigation characterized the peritumoral differences in fractional anisotropy (FA) and mean diffusivity (MD) values estimated from diffusion tensor imaging (DTI) in patients with HGGs and IM (19, 20). The higher FA and lower MD of HGGs in the peritumoral region were linked to a greater extent of infiltration of glioma upon the surrounding parenchyma (19). Traditional understanding of epileptogenic focus is related to structures in the temporal lobe. However, contemporary scientific advancements have established the specific anatomical basis of temporal-plus (TP+) epilepsy (21, 22). This new understanding has led researchers to scan outside the temporal lobe, toward neighboring regions for epileptogenic focus. Occipital, insular, or orbitofrontal areas are often considered for resection in addition to sections of the temporal lobe. Yet, limited literature exists on the etiological basis of extra-temporal involvement for seizure development (22). Although patients with brain tumors have not been directly linked to TP+ epilepsy, many tumor patients experience at least one seizure or develop multiple seizures even when the lesion is outside the temporal lobe. Therefore, investigating regions outside of the temporal lobe in brain tumor patients with seizures can be essential for future research.

Previously, our research team identified voxel-based metrics associated with a regional and global disruption in resting-state functional connectivity that may elucidate the epileptogenic focus and guide resection of cerebral cavernous malformations in patients with focal epilepsy (23). Despite single-institutional studies and observational cohorts utilizing administrative databases having identified the impact of seizures on short and long-term outcomes in patients undergoing intracranial tumor resection (4, 5, 24–27), meaningful clinical literature quantifying morphometric or volumetric analysis in tumor patients with seizures are limited. In the current investigation, we performed volumetric assessments to examine a wide range of anatomical regions [>100 regions of interests (ROIs)] across eight distinct cortical areas in patients with gliomas and IMs to elucidate the differences in cortical volume on the epileptogenicity of brain tumor. The primary objectives of the study are: (1) To quantify the normalized cortical volume estimates across predefined ROIs in tumor patients with and without seizures with respect to normal controls; and (2) To identify pertinent regions that express a significant contribution to differentiate seizure and non-seizure patients based upon volumetric distinctions. To achieve these objectives, we subjected the MR sequence images for tumor patients and controls to a battery of sophisticated brain-segmentation processing tools employing relevant BrainSuite and MATLAB modules. The study hypothesizes that the identification of significant volumetric differences across distinct ROIs in tumor patients could serve as possible biomarkers for predicting patients with the seizure disorder resulted from having brain tumors.

## Materials and Methods

### Study Protocol and Patient Population

In this retrospective design, adult patients (>18 years of age) with IM and gliomas that underwent surgical resection at the University Health/Louisiana State University Health Sciences Center (LSUHSC), Shreveport between January 2011 and June 2016 were identified. The preoperative surgical decision-making was not influenced by morphometric/volumetric imaging analysis, as performed in the current investigation, using a combination of BrainSuite and MATLAB custom designed modules; rather was guided by the consensus of a multi-disciplinary team of physicians in the institutional Tumor Board tailored upon patient's clinical characteristics and traditional neuroimaging modalities/scans. The study was approved by The LSUHSC Institutional Review before study initiation. Inclusion criteria applied for selection of patients that were surgically managed for their tumor pathology included: (1) confirmatory histopathological diagnoses, (2) complete MR sequences [1.5 Tesla; GE Medical Systems, Milwaukee, WI, USA] without evidence of movement artifacts. Image acquisition included T1-weighted Magnetization Prepared Rapid Acquisition with Gradient Echo (MPRAGE) sequences, diffusion tensor imaging (DTI), and 3-dimensional sagittal FLAIR sequences. Patients with a previous history of radiotherapy, chemotherapy, or neurosurgical intervention and those with multiple intracranial tumors (>2) were excluded. A review of medical records was conducted on eligible patients for the extraction of pertinent data on demographics (age, gender, race) and clinical characteristics. The latter included the presence of seizure at presentation, tumor-specific data including laterality, location, histology, and primaries for patients with IM. In addition to medical chart review, electroencephalogram (EEG) findings were used for seizure confirmation.

### Controls

Thirty-four healthy controls matched for age [range: 19–79 years] and gender [15 women] were included and served as a comparison group. All control subjects were free of any neurological diseases and had no prior history of any neurological diseases. Image acquisition and processing for the control subjects was performed in a manner like that of the eligible patients.

### Morphometric/Volumetric Analysis

**Figure 1:**
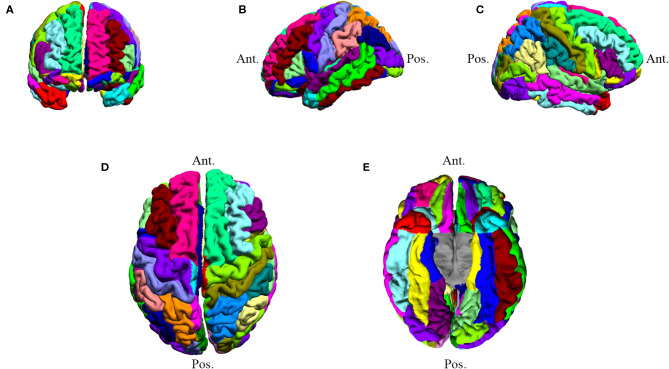
Subject DT007: BrainSuite processed MR image. The image underwent brain segmentation to determine COI values. DT007 was a 57-year-old male with a high-grade glioblastoma and presented with seizures. Colors are used to distinguish ROIs. **(A)** Anterior coronal view **(B)** Left hemisphere view **(C)** Right hemisphere view **(D)** Superior axial view **(E)** Inferior axial view.

For image processing, T1-MPRAGE sequences of all eligible patients (*n* = 102) and controls (*n* = 34) were subjected to the inbuilt automated cortical surface extraction processing in the BrainSuite software (version 18a; http://brainsuite.org/). This allows for stripping the skull from the MR sequences and initiates brain segmentation (Figure 1). Anatomical information from both the cortical surface models in the predefined atlas (BrainSuiteAtlas1) of BrainSuite and the volumetric estimates computed from stripped T1-MPRAGE images are utilized for co-registration between the patient imaging and the atlas (28–31). To compute relevant morphometric/volumetric data, partial tissue fraction volume was utilized along with co-registration, which yielded a single output file for each patient/control. The parcellation yielded volumes (or morphometrics) across 103 regions of interest (ROIs) using the Collin27 atlas within eight different cortical surface categories of interest (COIs): mean cortical thickness (mm), gray matter (GM) volume (mm3), cerebrospinal fluid volume (CSF) (mm3), white matter (WM) volume (mm3), total volume (mm3), mid cortical area (mm^2^), inner cortical area (mm^2^), and pial cortical area (mm^2^). (Figure 1) The total volume was the summation of WM and GM volume. Out of the 102 patients deemed eligible using the study criteria, ten were excluded with mutual consensus following a two-step independent review [A.E. and C.F.] owing to processing and segmentation errors encountered in BrainSuite.

### Volume Normalization and Z-Score Derivation

To minimize bias arising from variation in volume across patients due to differences in brain sizes/volume, whole-brain normalization was performed as a function of percent-volume for every ROI across the four cortical COIs [GM, CSF, WM, and total volume]. Quantified estimates of raw volume for ROIs were normalized in relation to individual patient/controls overall ROI. This was performed by computing percent-volume of each ROI by dividing individual ROI raw volume with summation of volumes across all ROIs for respective COIs factored by two (to accommodate both hemispheres). Following normalization, z-scores were computed for individual ROIs across all COIs. For COI's signifying area morphometrics [cortical areas: inner, mid, pial] or thickness [cortex], z-scores were computed from raw values for cortical areas and thickness, respectively. Prior to estimating z-scores, tumor patients were grouped based upon the presence of seizures, within each COI, in alignment with the study objective. Z-scores derivation for each ROI was performed using the following formula:

$$\begin{array}{l} \text{z}-\text{score}=\frac{Patient\;ROI\;Value\;-\;Control\;ROI\;\mu }{Control\;ROI\;\sigma } \end{array}$$

To exclude aberrant z-score values affecting estimates, the mean and standard deviation (SD) for each ROI within the seizure and non-seizure COI's were calculated. ROI values beyond three SD (top/bottom 0.135%) on either end of the z-score spectrum were excluded.

### Primary Outcome Measures

The primary outcome measures were: (1) mean z-score estimates for brain tumor patients with and without seizures and (2) ROI's associated with significant differences across z-score estimates between the two patient groups.

### Statistical Analysis

Categorical variables were expressed as a function of frequency and proportion while values of quantitative variables were reported as mean ± standard deviation (SD). The differences in categorical variables across tumor patients [gliomas, IM] and control were analyzed using Pearson's χ^2^ test of proportion or Fisher Exact test (32) as appropriate. The norm for analyzing differences in quantitative values across groups was based upon testing for Gaussian distribution. For analyzing differences across 2 groups [gliomas vs. IM; gliomas vs. controls; IM vs. controls], an independent-sample t-test or non-parametric Mann-Whitney *U*-test were utilized. For assessing differences in mean estimates across 3 groups [gliomas, IM and controls], one-way analysis of variance (ANOVA) or the non-parametric Kruskal-Wallis tests was employed.

The ROI's within individual COI's demonstrating significant one-tailed differences in z-scores were subjected to further analysis for feature weight determination within the seizure and non-seizure group using the MATLAB's Statistics and Machine Learning Toolbox. Serial iterations using the neighborhood component analysis (NCA) feature selection was utilized to determine the factor having the most weight in the classification algorithm's attempt to label each tumor patient based on the presence of seizure (vs. non-seizure) based on z-scores from the relevant COIs. To perform this, the cohort of tumor patients was split into a testing set (*n* = 66) and a training set (*n* = 26). Patients in both sets were assigned class labels as either seizure or non-seizure. More specifically, feature weights were computed using the MATLAB function for predictors and responses in which the predictors were the ROIs for every patient in each COI that was significant, and the response was the subject's seizure classification (coding for non-seizure = 0; seizure as 1). NCA was used to determine the patterns that classified each patient as either seizure or non-seizure and determined the component (ROI) that had the most weight on this classification by taking out each component iteratively until a maximum prediction accuracy was achieved (Tables S3, S4).

All statistical analyses were performed using SPSS version 25.0 (IBM Corp., NY), R Foundation for Statistical Computing (64-bit; version 3.3.3) and MATLAB. Unless otherwise stated (Tables S1, S2), all reported statistical estimates are derived from a 2-tailed significance set at a 5% alpha value.

## Results

Overall, 92 patients with brain tumors were included in the study. The mean age of the cohort was 58.0 ± 12.8 years, and 45% were women. Of these, ~55% (*n* = 51) had a low/high grade glioma while 45% (*n* = 41) had IM. In patients with gliomas and IMs, no statistical differences was noted in terms of age (56 vs. 60 years; *p* = 0.145), gender (women: 37 vs. 54%; *p* = 0.116), tumor laterality (right: 43.1 vs. 39%; *p* = 0.690) or location; however racial differences were observed with a higher proportion of whites presenting with gliomas compared to IMs (75 vs. 53%; *p* = 0.029). The overall prevalence of seizure in our cohort was 28%, with no statistical differences noted across patients with glioma and IMs (37 vs. 22%; *p* = 0.228). Nearly 86% (*n* = 44) of glioma patients had an HGG vs. 14% (*n* = 7) with LGG. In HGGs, astrocytomas (WHO Grade III or IV) constituted the majority of the cohort (*n* = 43; 84%). In patients with IMs, most had primary cancer in the lung (*n* = 29; 71%), usually of the poorly-differentiated subtype of the non-small cell lung cancer (NSCLC). This was followed by breast cancer (*n* = 7; 17.1%), lymphoma (*n* = 2; 4.9%) and one each (2.9%) for metastatic melanoma, endometrial cancer, and renal cell carcinoma (Table 1). An overview of demographics and clinical characteristics of patients and controls is presented in Table 1.

**Table 1:** Demographics and clinical characteristics of patients with brain tumors (gliomas and IM) with respect to controls.

**Characteristics**	**Gliomas**	**IM**	**Total**	***P* value**	**Controls**	***P* value**
	***N* = 51**	***N* = 41**	***N* = 92**		***N* = 34**	
**Age, years**						
Mean ± SD	56.3 ± 14.5	60.1± 10.2	58.0 ± 12.8	0.145	28.6 ± 10.7	**<0.001**
Median (IQR)	58 (25)	60 (10)	59 (14)		26 (7)	
Range	26-83	34-83	26-83		19-79	
**Gender, n (%)**						
Male	32 (62.7)	19 (46.3)	51 (55.4)	0.116	19 (55.9)	0.290
Female	19 (37.3)	22 (53.7)	41 (44.6)		15 (44.1)	
**Race**, ***n*** **(%)**						
Whites	38 (74.5)	21 (52.5)	59 (64.1)	**0.029**	-	-
African-Americans	12 (23.5)	19 (47.5)	31 (33.7)	**0.017**	-	-
Unknown	1 (2.0)	0 (0.0)	1 (1.1)	1.000^†^	-	-
**Tumor Laterality**, ***n*** **(%)**						
Right	22 (43.1)	16 (39.0)	38 (41.3)	0.690	-	-
Left	27 (52.9)	17 (41.5)	44 (47.8)	0.273	-	-
Corpus callosum	1 (2.0)	0 (0)	1 (1.1)	1.000^†^	-	-
Intraventricular^‡^	1 (2.0)	0 (0)	1 (1.1)	1.000^†^	-	-
Bilateral	0 (0)	8 (19.5)	8 (8.7)	**0.001**^†^	-	-
**Tumor Location**, ***n*** **(%)**						
Midline	5 (9.8)	1 (2.4)	6 (6.5)	0.220^†^	-	-
Temporal	17 (33.3)	9 (22.0)	26 (28.3)	0.228	-	-
Extra-temporal	29 (56.9)	29 (70.7)	58 (63.0)	0.171	-	-
Midline + Extra-temporal	0 (0)	(4.9)	2 (2.2)	0.196^†^	-	-
**Tumor Histology**, ***n*** **(%)**						
***High-grade gliomas***	**44 (86.2)**	-		-	-	-
Astrocytoma	43 (84.2)	-		-	-	-
Oligodendroglioma	1 (2.0)	-		-	-	-
***Low-grade gliomas***	**7 (13.8)**	-		-	-	-
Astrocytoma	3 (5.9)	-		-	-	-
Oligodendroglioma	3 (5.9)	-		-	-	-
Oligo-astrocytoma	1 (2.0)	-		-	-	-
**Primary Cancer**, ***n*** **(%)**						
Lung		29 (70.7)		-	-	-
Breast		7 (17.1)		-	-	-
Lymphoma		2 (4.9)		-	-	-
Melanoma		1 (2.4)		-	-	-
Endometrial adenocarcinoma		1 (2.4)		-	-	-
Renal cell		1 (2.4)		-	-	-
**Seizures**, ***n*** **(%)**	19 (37.3)	9 (21.9)	26 (28.3)	0.228	-	-

† *Fisher exact test*;

‡ *Included few patients with midline tumors*.

*The bold P-values depict significant statistical differences across the groups at Type I error set at 5%*.

A group of 34 gender-matched controls (44% women; 56% men; age range: 19–79 years) were included for comparison. Comparing controls to patients with glioma (*p* = 0.527) or IMs (*p* = 0.411), no gender differences were noted.

### Z-Score Estimates Across ROI's

**Figure 2:**
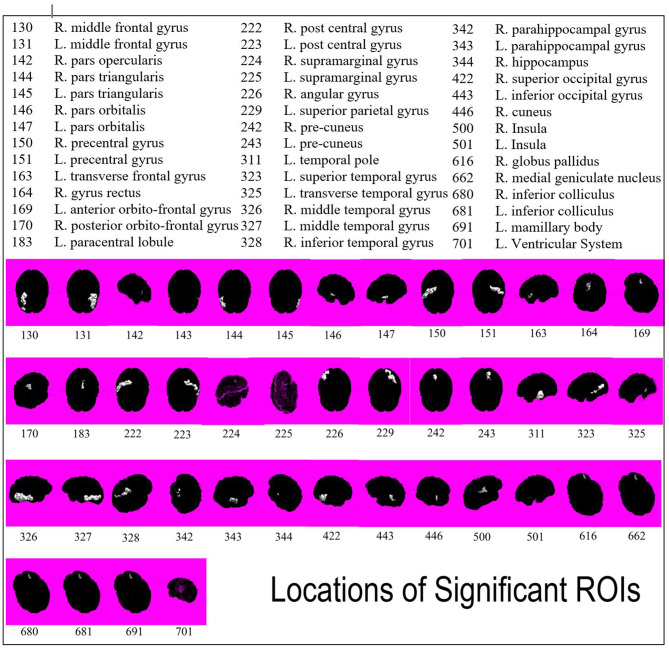
Spatial representation of all forty-two ROIs found to be significant in differentiating seizure and non-seizure brain tumor patients. ROIs are indicated by white highlight and numbers are associated by key.

**Figure 3:**
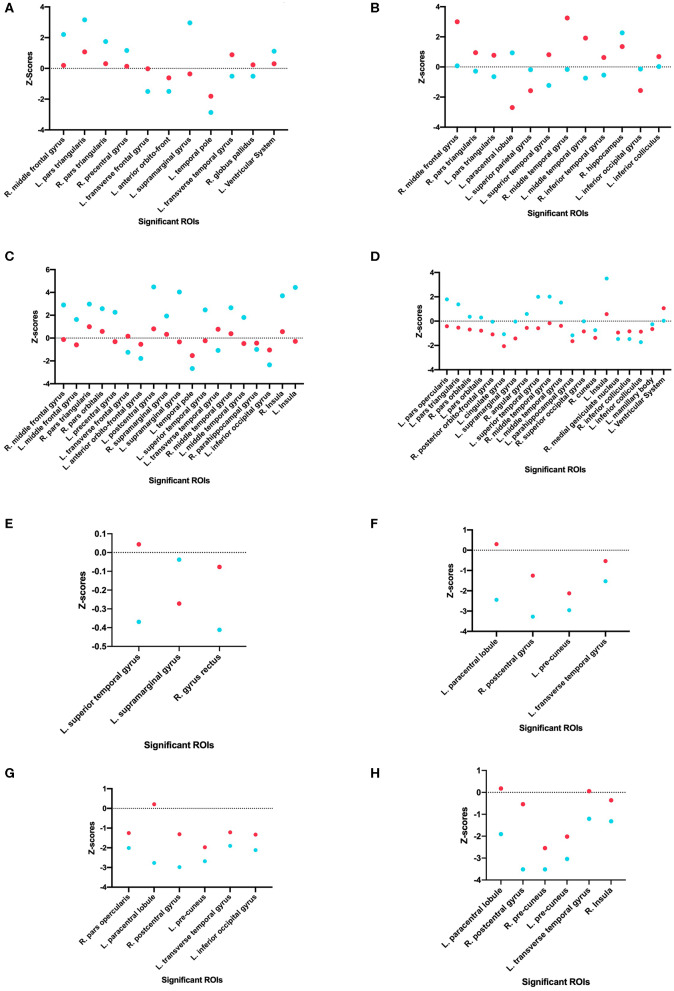
Comparison of seizure and non-seizure subject groups, in terms of z-scores, for each significant ROI among the eight different categories of interest. The points with a more positive value indicate a raw score that is higher than the control, while points with a more negative value indicate a raw score that is lower than the control. Each graph indicates a different COI: **(A)** Gray matter volume, **(B)** White matter volume, **(C)** Total matter volume, **(D)** CSF volume, **(E)** Cortical thickness, **(F)** Mid cortical area, **(G)** Inner cortical area, and **(H)** Pial cortical area.

Using one-tailed significance testing, forty-two ROIs among eight categories of interest (COIs) were identified to have significant differences in z-score estimates. Spatial orientation of these potential ROIs that differ based upon normalized volume [GM. WM, total volume and CSF], area [cortical: inner, mid and pial] and cortical thickness across COIs are depicted in Figure 2. The mean z-score estimates for these ROIs across individual COI's depicting one-tailed significance are plotted in a dot-diagram for seizure vs. non-seizure (Figures 3A–H). Also, the quantified z-score estimates across tumor patients' groups upon the presence of seizure are tabulated (Tables S1, S2).

### Volumetric Morphometric Assessments

The filtering of ROI's significant on one-tailed significance was performed by assessments using two-tailed testing for GM, WM, Total, and CSF volume. The two-tailed significance testing formed the primary basis for identifying true differences across seizure and non-seizure patients and presented in Tables 2, 3 for volumetric and morphometric (area and thickness) differences across different ROIs for the 8 COIs, respectively.

**Table 2:** Significant differences in the mean z-scores in tumor patients with seizure vs. non-seizure across all volumetric COIs using two-tailed significance.

**Significant ROI's**	**Gray matter**			**White matter**			**Total volume**			**CSF volume**		
	**Seizure**	**Non-seizure**	***P* value (2-tailed)**	**Seizure**	**Non-seizure**	***P* value (2-tailed)**	**seizure**	**Non-seizure**	***P* value (2-tailed)**	**seizure**	**Non-seizure**	***P* value (2-tailed)**
R. middle frontal gyrus	-	-	-	3.00	0.07	**0.019**	2.90	−0.12	**0.011**	-	-	-
L. pars opercularis	-	-	-	-	-	-	-	-	-	1.79	−0.42	**0.022**
L. pars triangularis	-	-	-	-	-	-	-	-	-	1.38	−0.54	**0.001**
R. pars orbitalis	**1.75**	**0.31**	**0.094**	-	-	-	**2.58**	**0.59**	**0.055**	**0.36**	**−0.69**	**0.009**
L. pars orbitalis	-	-	-	0.77	−0.64	**0.042**	-	-	-	0.29	−0.78	**0.003**
L. transverse frontal gyrus	−1.50	−0.02	**0.010**	-	-	-	−1.25	0.16	**0.017**	-	-	-
R. posterior orbito-frontal gyrus	-	-	-	-	-	-	-	-	-	−0.05	−1.09	**0.048**
L. paracentral lobule	-	-	-	−2.70	0.94	**0.040**	-	-	-	-	-	-
L. post-central gyrus	-	-	-	-	-	-	4.48	0.81	**0.017**	-	-	-
L. supramarginal gyrus	2.96	−0.36	**0.001**	-	-	-	4.04	−0.33	**0.002**	-	-	-
L. superior temporal gyrus	-	-	-	0.81	−1.23	**0.013**	2.47	−0.23	**0.013**	1.999	−0.583	**0.001**
L. transverse temporal gyrus	-	-	-	-	-	-	−1.08	0.77	**0.017**	-	-	-
R. middle temporal gyrus	-	-	-	**3.25**	**−0.17**	**0.008**	-	-	-	2.01	−0.17	**0.008**
L. middle temporal gyrus	-	-	-	1.92	−0.74	**0.008**	-	-	-	1.53	−0.39	**0.024**
L. inferior occipital gyrus	-	-	-	1.56	−0.14	**0.040**	-	-	-	-	-	-
L. Insula	-	-	-	-	-	-	4.43	0.01	**0.047**	3.51	0.58	**0.014**
L. inferior colliculus	-	-	-	-	-	-	-	-	-	−1.73	−0.87	**0.010**
L. Ventricular System	1.12	0.30	**0.016**	-	-	-	-	-	-	-	-	-

*Yellow highlighted values represent the ROI in respective COI to have the highest weight in the NCA. The bold P-values depict significant statistical differences across the groups at Type I error set at 5%*.

**Table 3:** Mean differences in z-scores across Cortical Thickness and Cortical Area zones (mid, inner, and pial) across tumor patients with and without seizures using two-tailed significance.

**Significant ROI's**	**Cortical thickness**			**Mid cortical area**			**Inner cortical area**			**Pial cortical area**		
	**Seizure**	**Non-seizure**	***P* value (2-tailed)**	**Seizure**	**Non-seizure**	***P* value (2-tailed)**	**Seizure**	**Non-seizure**	***P* value (2-tailed)**	**Seizure**	**Non-seizure**	***P* value (2-tailed)**
L. paracentral lobule	-	-	-	-	-	-	−2.77	0.21	0.022	-	-	-
R. post-central gyrus	-	-	-	-	-	-	−2.98	−1.31	0.047	-	-	-
L. supramarginal gyrus	**0.33**	**−0.13**	**0.055**	-	-	-	-	-	-	**-**	-	-
L. pre-cuneus	-	-	-	-	-	-	**−2.68**	**−1.97**	**0.069**	-	-	-
L. superior temporal gyrus	−0.41	0.09	**0.020**	-	-	-	-	-	-	-	-	**-**
L. transverse temporal gyrus	-	-	-	**−1.53**	**−0.53**	**0.007**	-	-	-	**−1.21**	**0.06**	**0.005**

*Yellow highlighted values represent the ROI in respective COI to have the highest weight in the NCA*.

*The bold P-values depict significant statistical differences across the groups at Type I error set at 5%*.

### Feature Selection: Neighborhood Component Analysis

Using feature selection, the ROIs with the most influence on differentiating the seizure and non-seizure groups in tumor patients was found for all eight COIs (Figure S1). The NCA analysis demonstrated the normalized volume of right-sided pars orbitalis across three COIs, viz. GM, total volume and CSF volume, had the most weight in differentiating tumor patients with seizure from non-seizures. Analysis of the COI involving the WM, right-sided middle temporal gyrus was noted to have the most weight in distinguishing the presence of seizures from non-seizures.

Left-sided transverse temporal gyrus, left precuneus, left transverse temporal, and left supramarginal gyrus were associated with having the highest weight in the differentiation of seizure vs. non-seizure in tumor patients for morphometrics relating to cortical areas in the pial, inner and mid regions and cortical thickness, respectively.

## Discussion

In this study, we utilized image processing techniques to analyze MR sequences of patients with low/high-grade gliomas and IMs, as well as a healthy group of controls. By categorizing tumor patients into two groups, seizure and non-seizure, we employed multiple metrics to differentiate the two groups across eight predefined COIs in the brain: gray matter volume, white matter volume, total matter volume, CSF volume, cortical thickness, middle cortical area, inner cortical area, and pial cortical area.

The study findings suggest significant difference in z-scores in tumor patients with seizure vs. those without seizures across several temporal and extra-temporal regions. Most consistent extratemporal areas that demonstrated significant differences in z-scores across seizure vs. non-seizure patients were right pars orbitalis, left supramarginal gyrus, left transverse gyrus, right-middle frontal gyrus and left paracentral lobule. Right-sided pars orbitalis, the rostral portion of the inferior frontal gyrus, has been implicated in high-frequency oscillations during focal neocortical seizures, especially in patients with drug-resistant epilepsy (33). The paracentral lobule, spanning over the fronto-parietal lobe, has been termed an “independent pro-epileptogenic factor” in relation to primary brain tumors due to the high volume of neurons in the structure and its association with the primary motor cortex (34). These results support the claim that the frontal lobe influences epileptogenesis progression, especially among tumor patients of all types and locations (34). Specifically, ictal spikes in the paracentral lobule from the non-dominant hemisphere are characterized by sexual sensations affecting the genitalia (35). Unfortunately, given the scope of the current study, we could not explore the relationship between tumor location and seizure semiology. Other frontal lobe structures that are known to be associated with epileptic loci, significant in our analysis, include middle frontal gyrus, pars opercularis and pars triangularis. Another pertinent ROI, the supramarginal gyrus, has been implicated in TP+ epilepsy syndromes both in patients with and without tumors (22), and differed significantly across tumor patients with seizures vs. those without seizure activity. While it is not surprising regarding the association of temporal structures (middle and superior temporal gyrus, transverse temporal gyrus) in epilepsy, studies have shown that temporal lobe epilepsy might be due to the dysfunction of GABA-B receptors (2, 36).

Our second method of determining which ROIs had the most weight in predicting whether a patient would present with seizures was Neighborhood Component Analysis (NCA) Feature Selection. NCA was used to determine the patterns that classified each subject as either seizure or non-seizure and determined the component (ROI) that had the most weight on this classification by taking out each component iteratively until a maximum prediction accuracy was achieved. This method determined that the ROIs—L. supramarginal gyrus and L. transverse temporal gyrus, along with three other ROIs—appeared again as an accurate classifier as to whether the patient was in the seizure or non-seizure groups. The COIs that these ROIs appeared in were Cortical Thickness (L. supramarginal gyrus) and Mid Cortical Area (L. transverse temporal gyrus). The reappearance of these ROIs' significance supports the claim that they have an important role in differentiating seizure and non-seizure patients. In addition to these regions of interest, the R. pars orbitalis was found to have the most feature weight in three categories of interest: gray matter volume, total volume, and CSF volume. Because of this unexpected finding, as this region of interest is not normally associated with seizure activity, it provides an opportunity for further research on this subject to confirm or deny any suspicions.

Although it is beneficial to elucidate ROI's associated with seizure development in tumor patients, the authors acknowledge that tumor location alone cannot definitively predict seizures. Although temporal lobe tumors have higher proponderence for developing seizures but not all such tumors result in seizures. Pathological predisposition for seizures in tumors arising from the temporal area can be explained from dual reasoning: hippocampal sclerosis and/or co-existent focal cortical dysplasia in temporal or extratemporal structures. On the contrary, the authors believe that such associations for extra-temporal tumors are harder to deduce. Compared to deep-seated or infratentorial tumors, superficially located tumors are more likely to be seizure prone due to its proximity to the neuronal cell bodies and thereby increasing the likelihood of cortical irritation (37, 38). Further, the literature suggests that epileptogenicity of frontal or parietal lobe tumors is second to that of temporal lobe tumors followed by occipital lobe lesions which are considered least epileptogenic (37, 38). The identified extra-temporal ROIs in our study can explain the association of fronto-parietal epilepsies. In relation to the occurrence of intraoperative seizures in tumor patients undergoing awake craniotomy, Gonen et al. concluded that tumors localized in the supplementary motor area (SMA) had higher incidence (OR: 11.36; *p* < 0.002) compared to non-SMA frontal, temporal, or parietal regions (39). The authors opine that the existing knowledge linking an association of tumor location with seizure propensity is derived from limited observational studies, and that a systematic review/meta-analysis using larger, granular, homogenous cohorts from the published literature is appreciable for strengthening such association.

## Study Limitations

Despite the merit of the current investigation, pertinent limitations governing the study need to be addressed. First, the heterogeneity in tumor types (subgroups of gliomas and IMs) and relatively smaller sample sizes within each group limit generalization of our findings owing to suboptimal power for subgroup analysis for tumor histology. Second, the study did not account for seizure semiology and/or localization of the epileptogenic zone with tumor location. However, given the focus of the investigation, quantifying and contrasting z-scores for ROIs across seizure and non-seizure brains would not be impacted due to this limitation. Regarding the study design, the retrospective, observational nature of our investigation fails to establish a causal relationship between the differences in the ROI's due to epileptogenic loci or a result of seizure spread, which could serve as a future direction for research on the topic. As the study was not powered enough, the lack of predictive parameters (e.g., sensitivity, specificity) limits validation of the ROIs for seizure prediction. Despite these limitations, the utility of the current investigation lies in the widespread brain areas that were examined to assess differences across scans of tumor patients with and without seizures. Discovering these ROIs in connection with differentiating seizure and non-seizure brain tumor patients has provided a foundation for more extensive research on this subject. With a larger cohort of patients, we suspect that among these significant regions found in this study, a smaller variety of ROIs will become solidified and provide a more specific connection to seizures, brain tumors, and the volume of structures in the brain.

## Conclusion

Our study elucidates potential morphological biomarkers for seizure targeting in patients with gliomas and IMs based upon morphometric and volumetric assessments. Amongst the widespread brain regions examined in our cohort, pars orbitalis, supramarginal andtemporal gyrus (middle, transverse), and the precuneus contribute a maximal potential for differentiation of seizure patients from non-seizure. The significance of these regions of interest using a *t*-test as well as feature selection, supports the claim that these areas are connected to tumoral seizures. In the future, gathering a larger cohort that specifies in a smaller variety of tumor types will be beneficial to this specific field of interest.

## Data Availability Statement

Datasets are available on request due to privacy/ethical restrictions. Relevant data has been posted/provided in the online Supplementary Material.

## Ethics Statement

The studies involving human participants were reviewed and approved by Louisiana State University, Shreveport. Written informed consent for participation was not required for this study in accordance with the national legislation and the institutional requirements.

## Author Contributions

HS, PK, AE, and CL contributed to study conceptualization and research design. AE and CF performed data acquisition/collection. AE, PK, CF, and KS performed data processing (analysis), statistical analysis, and interpretation. PK, AE, CF, and KS drafted the article. PK, KS, TH, and HS critically revised the article. All authors have read and approved the final version of the manuscript. CL, EG-T, AN, and HS provided administrative/technical/material support. Study supervision involved HS, CL, PK, and AE.

## Conflict of Interest

The authors declare that the research was conducted in the absence of any commercial or financial relationships that could be construed as a potential conflict of interest.
