# Supplementary material for: Biomarkers of Seizure Activity in Patients With Intracranial Metastases and Gliomas: A Wide Range Study of Correlated Regions of Interest
Source: Front Neurol. 2020 May 29;11:444. doi: 10.3389/fneur.2020.00444 (PMC7273506; doi:10.3389/fneur.2020.00444)
Supplement: Supplementary file 1 [file Data_Sheet_1.pdf]

Online Supplement

Biomarkers of Seizure Activity in Patients with Intracranial Metastasis and Gliomas: A Wide Range Study of Correlated Regions of Interest

*[1]Piyush Kalakoti, *[1]Alicia Edwards, [1]Christopher Ferrier, [2]Kanika Sharma, [3,4]Trong Huynh, [1]Christina Ledbetter, [5]Eduardo Gonzalez Toledo, [3,4]Anil Nanda, [3,4]Hai Sun

[1]Department of Neurosurgery, Louisiana State University Health Science Center, Shreveport, LA 71103, United States
[2]Neurology, University of Iowa Hospitals and Clinics, Iowa City, Iowa 52241, United States
[3]Department of Neurosurgery, Robert Wood Johnson Medical School, New Brunswick, NJ 08903, United States
[4]Department of Neurosurgery, Rutgers University, Newark, NJ 07103, United States
[5]Neuroradiology, Department of Radiology, Louisiana State University Health Science Center, Shreveport, LA 71103, United States

*Authors contributed equally to work and are co-primary authors

Keywords:
gliomas; intracranial metastases; seizures; pars orbitalis; supramarginal gyrus; pre-cuneus; BrainSuite, temporal plus epilepsy

Study Funding:
None

Conflicts of Interests/Disclosures:
None

Table of Contents

Supplementary Figures

Figure S1: Feature Selection Scatterplots for Each COI……………………………………………………………………………………………………3-4

Supplementary Tables

Table S1: Volume with one-tailed P Values………5-6

Table S2: Cortical Thickness and Area z-scores with one-tailed P values……………………………………………………………………………………7

Table S3: Feature Weights by ROI Number and Category of Interest……………………………………………………………………………………8-10

Table S4: Corresponding Indices for Feature Weight Graphs (Figure S1) for every COI………………………………………………………………11-12

Figure S1: Feature weight graphs for every COI using significant ROIs for each COI. ROI Index corresponds to index specified in **Table S4**

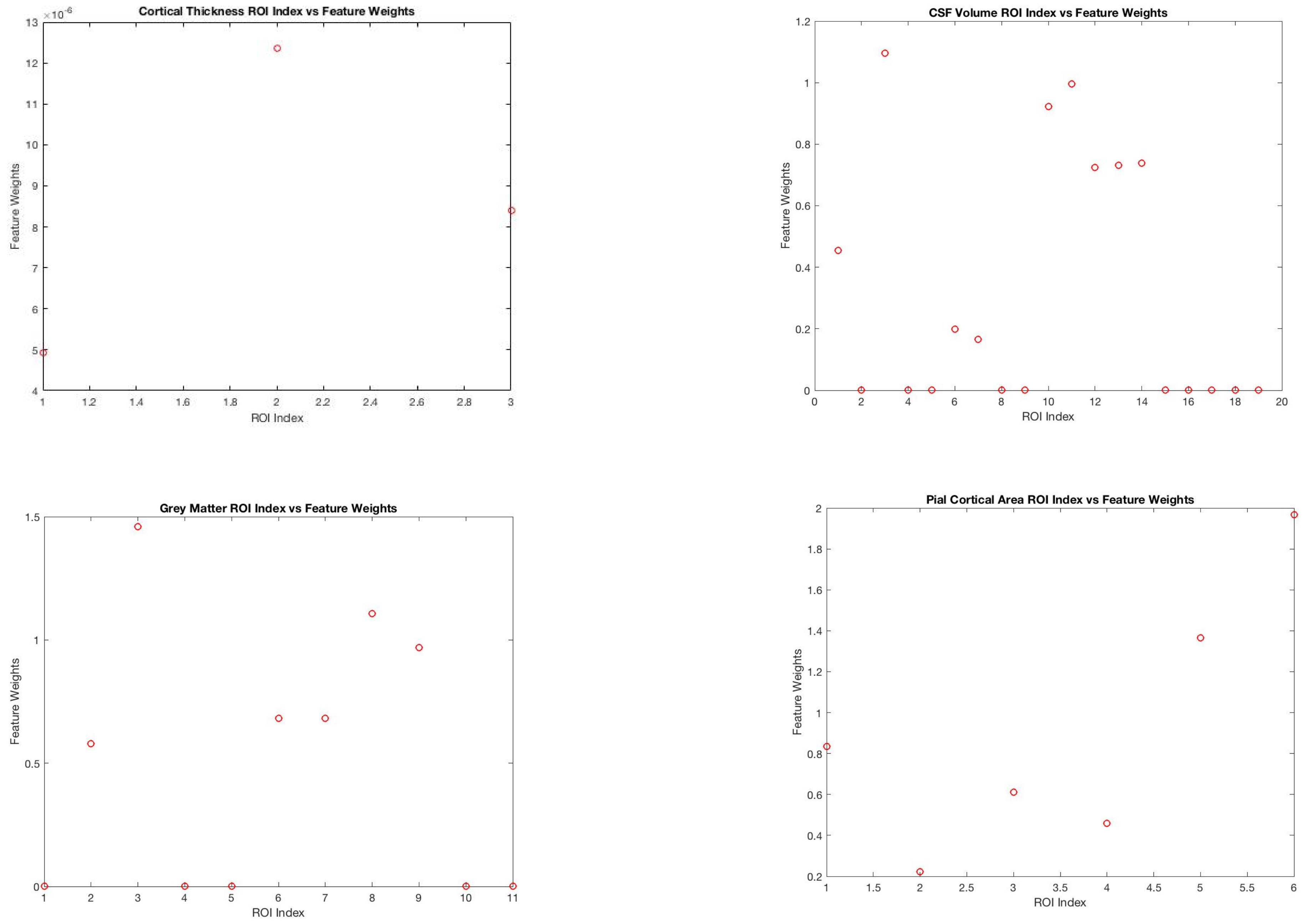

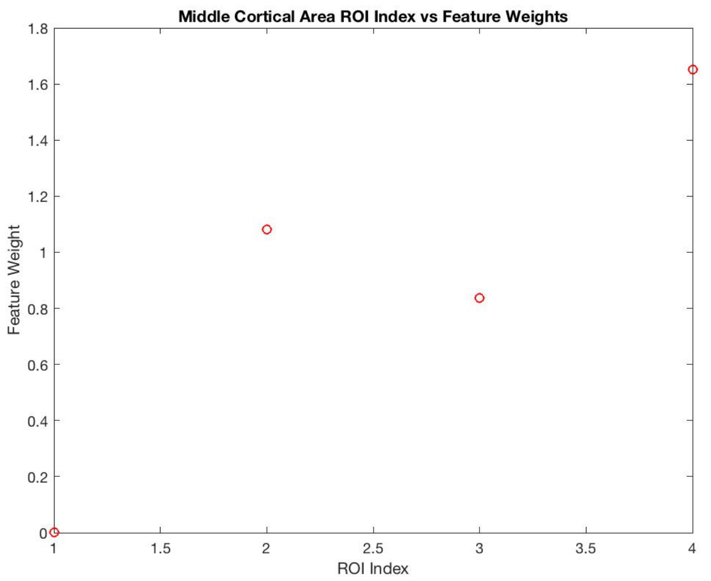
Middle Cortical Area ROI Index vs Feature Weights
Feature Weight
ROI Index

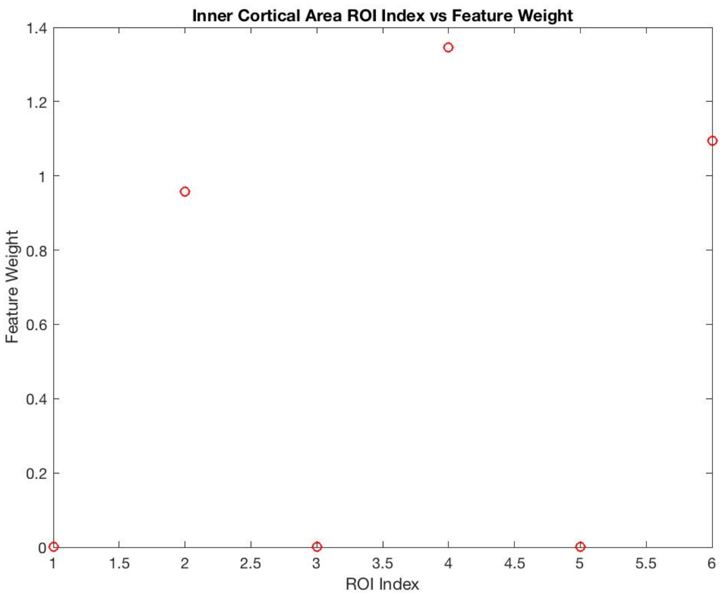
Inner Cortical Area ROI Index vs Feature Weight
Feature Weight
ROI Index

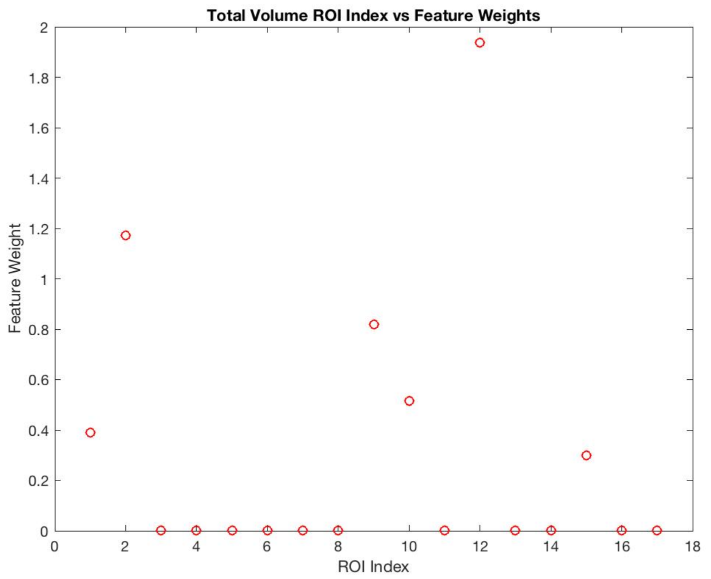
Total Volume ROI Index vs Feature Weights
Feature Weight
ROI Index

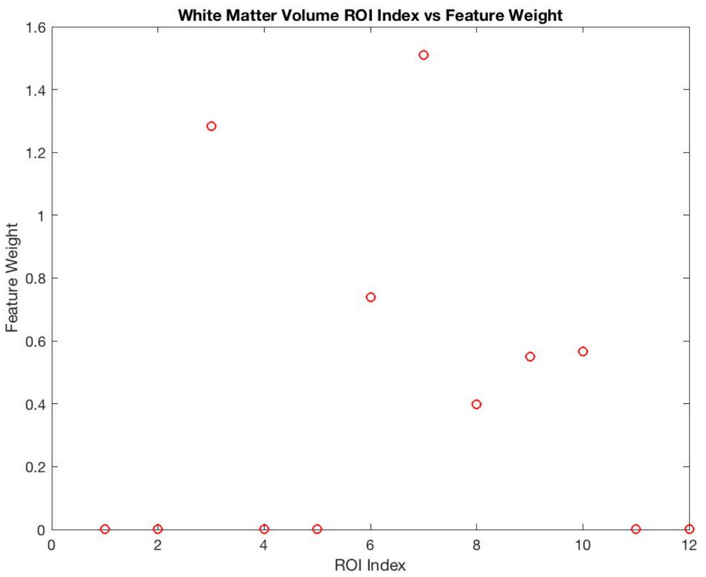
White Matter Volume ROI Index vs Feature Weight
Feature Weight
ROI Index

Table S1: Significant differences in the mean z-scores in tumor patients with seizure versus non-seizure across all volumetric COIs using one-tailed significance

Significant ROI's	Grey Matter			White Matter			Total Volume			CSF Volume		
	Seizure	Non-Seizure	*P* value † (1-tailed)	Seizure	Non-Seizure	*P* value † (1- tailed)	Seizure	Non-Seizure	*P* value † (1- tailed)	Seizure	Non-Seizure	*P* value † (1- tailed)
R. middle frontal gyrus	2.21	0.20	**0.045**	3.00	0.07	**0.009**	2.90	-0.12	**0.005**	-	-	-
L. middle frontal gyrus	-	-	-	-	-	-	1.63	-0.59	**0.037**	-	-	-
L. pars opercularis	-	-	-	-	-	-	-	-	-	1.79	-0.42	**0.011**
R. pars triangularis	-	-	-	0.95	-0.28	**0.042**	2.98	1.00	**0.049**	-	-	-
L. pars triangularis	3.16	1.08	**0.040**	-	-	-	-	-	-	1.38	-0.54	**0.001**
R. pars orbitalis	**1.75**	**0.31**	**0.047**	-	-	-	**2.58**	**0.59**	**0.027**	**0.36**	**-0.69**	**0.004**
L. pars orbitalis	-	-	-	0.77	-0.64	**0.021**	-	-	-	0.29	-0.78	0.002
R. precentral gyrus	1.17	0.14	**0.043**	-	-	-	-	-	-	-	-	-
L. transvers frontal gyrus	-1.50	-0.02	**0.005**	-	-	-	-1.25	0.16	**0.009**	-	-	-
L. anterior orbito-frontal gyrus	-1.50	-0.62	**0.027**	-	-	-	-1.78	-0.54	**0.041**	-	-	-
R. posterior orbito-frontal gyrus	-	-	-	-	-	-	-	-	-	-0.05	-1.09	**0.024**
L. cingulate gyrus	-	-	-	-	-	-	-	-	-	-1.07	-2.06	**0.030**
L. paracentral lobule	-	-	-	-2.70	0.94	**0.020**	-	-	-	-	-	-
L. post-central gyrus	-	-	-	-	-	-	4.48	0.81	**0.008**	-	-	-
L. supramarginal gyrus	2.96	-0.36	**<0.001**	-	-	-	4.04	-0.33	**0.001**	-0.03	1.43	**0.037**
R. angular gyrus	-	-	-	-	-	-	-	-	-	0.59	-0.56	**0.047**

L. superior parietal gyrus	-	-	-	-1.58	-0.18	**0.032**	-	-	-	-	-	-
L. temporal pole	-2.87	-1.81	**0.043**	-	-	-	-2.67	-1.54	**0.043**	-	-	-
L. superior temporal gyrus	-	-	-	0.81	-1.23	**0.007**	2.47	-0.23	**0.006**	2.00	-0.58	**<0.001**
L. transverse temporal gyrus	-0.51	0.89	**0.032**	-	-	-	-1.08	0.77	**0.009**	-	-	-
R. middle temporal gyrus	-	-	-	**3.25**	**-0.17**	**0.004**	2.66	0.38	**0.037**	2.01	-0.17	**0.004**
L. middle temporal gyrus	-	-	-	1.92	-0.74	**0.004**	0.81	-0.23	**0.038**	1.53	-0.39	**0.012**
R. inferior temporal gyrus	-	-	-	0.63	-0.54	**0.047**	-	-	-	-	-	-
L. parahippo-campal gyrus	-	-	-	-	-	-	-0.99	-0.44	**0.041**	-1.17	-1.65	**0.044**
R. hippocampus	-	-	-	1.36	2.27	**0.027**	-	-	-	-	-	-
R. superior occipital gyrus	-	-	-	-	-	-	-	-	-	-0.01	-0.85	**0.039**
L. inferior occipital gyrus	-	-	-	1.56	-0.14	**0.047**	2.34	-1.04	**0.033**	-	-	-
R. cuneus	-	-	-	-	-	-	-	-	-	-0.74	-1.38	**0.041**
R. Insula	-	-	-	-	-	-	3.70	0.80	**0.049**	-	-	-
L. Insula	-	-	-	-	-	-	4.43	0.50	**0.023**	3.51	0.58	**0.007**
R. globus pallidus	-0.51	0.23	**0.029**	-	-	-	-	-	-	-	-	-
R. medial geniculate nucleus	-	-	-	-	-	-	-	-	-	-1.47	-0.94	**0.031**
R. inferior colliculus	-	-	-	-	-	-	-	-	-	-1.48	-0.84	**0.035**
L. inferior colliculus	-	-	-	0.69	0.02	**0.040**	-	-	-	-1.73	-0.87	**0.005**
L. mamillary body	-	-	-	-	-	-	-	-	-	-0.26	-0.65	**0.049**
L. Ventricular System	1.12	0.30	**0.008**	-	-	-	-	-	-	0.03	1.06	**0.026**

†Yellow highlighted values represent the ROI in respective COI to have the highest weight in the NCA

Table S2: Mean differences in z-scores across Cortical Thickness and Cortical Area zones (mid, inner and pial) across tumor patients with and without seizures

Significant ROI's	Cortical Thickness			Mid Cortical Area			Inner Cortical Area			Pial Cortical Area		
	Seizure	Non-Seizure	*P* value † (1-tailed)	Seizure	Non-Seizure	*P* value † (1- tailed)	Seizure	Non-Seizure	*P* value † (1- tailed)	Seizure	Non-Seizure	*P* value † (1- tailed)
R. pars opercularis	-	-	-	-	-	-	-2.01	-1.25	**0.049**	-	-	-
R. gyrus rectus	-0.34	0.12	**0.027**	-	-	-	-	-	-	-	-	-
L. paracentral lobule				-2.44	0.30	**0.027**	-2.77	0.21	**0.011**	-1.91	0.18	**0.043**
R. postcentral gyrus	-	-	-	-3.27	-1.25	**0.029**	-2.98	-1.31	**0.024**	-3.51	-0.54	**0.032**
L. supramarginal gyrus	**0.33**	**-0.13**	**0.028**									
R. pre-cuneus				-	-	-	-	-	-	-3.51	-2.55	**0.027**
L. pre-cuneus	-	-	-	-2.96	-2.12	**0.039**	**-2.68**	**-1.97**	**0.035**	-3.04	-2.02	**0.043**
L. superior temporal gyrus	-	-	-	-	-	-	-	-	-			
L. transverse temporal gyrus	-0.41	0.09	**0.010**	**-1.53**	**-0.53**	**0.004**	-1.90	-1.21	**0.034**	**-1.21**	**0.06**	**0.003**
R. insula				-	-	-	-	-	-	-1.32	-0.36	**0.028**
L. inferior occipital gyrus	-	-	-				-2.12	-1.33	**0.046**			

†Yellow highlighted values represent the ROI in respective COI to have the highest weight in the NCA

Table S3: Feature Weights By ROI Number and Category of Interest

ROI Number	Total Volume	Grey Matter	White Matter	CSF Volume	Cortical Pial Area	Cortical Middle Area	Cortical Inner Area	Cortical Thickness	ROI Number
130	3.89E-01	8.71E-06	6.04E-06	-	-	-	-	-	130
131	1.17E+00	-	-	-	-	-	-		131
142	-	-		-	-	-	1.32E-06	-	142
143	-	-	-	4.55E-01	-	-	-	-	143
144	2.34E-06	-	3.61E-06	-	-	-	-	-	144
145	-	5.80E-01	-	3.08E-06	-	-	-	-	145
146	1.89E-06	1.46E+00	-	1.10E+00	-	-	-	-	146
147	-	-	1.28E+00	9.44E-06	-	-	-	-	147
150	-	2.92E-06	-	-	-	-	-	-	150
163	2.44E-07	2.00E-05	-	-	-	-	-	-	163
164	-	-	-	-	-	-	-	4.92E-06	164
169	8.30E-07	6.82E-01	-	-	-	-	-	-	169
170	-	-	-	8.89E-06	-	-	-	-	170
183	-	-	3.31E-06		8.35E-01	4.35E-08	9.57E-01	-	183
185	-	-	-	1.99E-01	-	-	-	-	185
222	-	-	-	-	2.22E-01	1.08E+00	1.50E-06	-	222
223	1.16E-06	-	-	-	-	-	-	-	223

225	*6.84E-07*	*6.82E-01*	-	*1.65E-01*	-	-	-	*1.23E-05*	*225*
226	-	-	-	*1.48E-05*	-	-	-	-	*226*
229	-	-	*3.67E-06*	-	-	-	-	-	*229*
242	-	-	-	-	*6.10E-01*	-	-	-	*242*
243	-	-	-	-	*4.59E-01*	*8.38E-01*	*1.35E+00*	-	*243*
311	*8.20E-01*	*1.11E+00*	-	-	-	-	-	-	*311*
323	*5.16E-01*	-	*7.40E-01*	*1.89E-06*	-	-	-	*8.40E-06*	*323*
325	*5.56E-06*	*9.70E-01*	-	-	*1.37E+00*	*1.65E+00*	*8.04E-07*	-	*325*
326	*1.94E+00*	-	*1.51E+00*	*9.23E-01*	-	-	-	-	*326*
327	*2.36E-06*	-	*3.99E-01*	*9.96E-01*	-	-	-	-	*327*
328	-	-	*5.51E-01*	-	-	-	-	-	*328*
342	*1.05E-06*	-	-	-	-	-	-	-	*342*
343	-	-	-	*7.24E-01*	-	-	-	-	*343*
344	-	-	*5.67E-01*	-	-	-	-	-	*344*
422	-		-	*7.31E-01*	-	-	-	-	*422*
443	*3.00E-01*	-	*1.99E-06*	-	-	-	*1.09E+00*	-	*443*
446	-	-	-	*7.38E-01*	-	-	-	-	*446*
500	*1.31E-06*	-	-	-	*1.97E+00*	-	-	-	*500*
501	*5.30E-07*	-	-	*6.98E-06*	-	-	-	-	*501*
616	-	*4.96E-07*	-	-	-	-	-	-	*616*
662	-	-	-	*2.51E-05*	-	-	-	-	*662*

680	-	-	-	*1.38E-05*	-	-	-	-	*680*
681	-	-	*2.78E-06*	*5.29E-06*	-	-	-	-	*681*
691	-	-	-	*7.98E-06*	-	-	-	-	*691*
701	-	*6.77E-07*	-	-	-	-	-	-	*701T*

Values not included for a category of interest for any given ROI indicates that the ROI for that category of interest was not found to be significant and therefore was not included in NCA . See index table in main manuscript for ROI description

Table S4: Corresponding Indices for Feature Weight Graphs (Figure S1) for every COI								
	Indices							
**ROI**	**Total Volume**	**Grey Matter**	**White Matter**	**CSF Volume**	**Cortical Pial Area**	**Cortical Middle Area**	**Cortical Inner Area**	**Cortical Thickness**
**130**	1	1	1	-	-	-	-	-
**131**	2	-	-	-	-	-	-	-
**142**	-	-	-	-	-	-	1	-
**143**	-	-	-	1	-	-	-	-
**144**	3	-	2	-	-	-	-	-
**145**	-	2	-	2	-	-	-	-
**146**	4	3	-	3	-	-	-	-
**147**	-	-	3	4	-	-	-	-
**150**	-	4	-	-	-	-	-	-
**163**	5	5	-	-	-	-	-	-
**164**	-	-	-	-	-	-	-	1
**169**	6	6	-	-	-	-	-	-
**170**	-	-	-	5	-	-	-	-
**183**	-	-	4	-	1	1	2	-
**185**	-	-	-	6	-	-	-	-
**222**	-	-	-	-	2	2	3	-
**223**	7	-	-	-	-	-	-	-
**225**	8	7	-	7	-	-	-	2
**226**	-	-	-	8	-	-	-	-
**229**	-	-	5	-	-	-	-	-
**242**	-	-	-	-	3	-	-	-
**243**	-	-	-	-	4	3	4	-

**311**	9	8	-	-	-	-	-	-
**323**	10	-	6	9	-	-	-	3
**325**	11	9	-	-	5	4	5	-
**326**	12	-	7	10	-	-	-	-
**327**	13	-	8	11	-	-	-	-
**328**	-	-	9	-	-	-	-	-
**342**	14	-	-	-	-	-	-	-
**343**	-	-	-	12	-	-	-	-
**344**	-	-	10	-	-	-	-	-
**422**	-	-	-	13	-	-	-	-
**443**	15	-	11	-	-	-	6	-
**446**	-	-	-	14	-	-	-	-
**500**	16	-	-	-	6	-	-	-
**501**	17	-	-	15	-	-	-	-
**616**	-	10	-	-	-	-	-	-
**662**	-	-	-	16	-	-	-	-
**680**	-	-	-	17	-	-	-	-
**681**	-	-	12	18	-	-	-	-
**691**	-	-	-	19	-	-	-	-
**701**	-	11	-	-	-	-	-	-
